# MafB Maintains *β*-Cell Identity under MafA-Deficient Conditions

**DOI:** 10.1128/mcb.00541-21

**Published:** 2022-07-11

**Authors:** Zhaobin Deng, Akihiro Kuno, Masami Ojima, Satoru Takahashi

**Affiliations:** a Department of Anatomy and Embryology, Faculty of Medicine, University of Tsukuba, Ibaraki, Japan; b School of Comprehensive Human Sciences, Doctoral Program in Biomedical Sciences, University of Tsukuba, Ibaraki, Japan; c PhD Program in Human Biology, School of Integrative and Global Majors, University of Tsukuba, Ibaraki, Japan; d Laboratory Animal Resource Center (LARC), University of Tsukuba, Ibaraki, Japan; e Life Science Center, Tsukuba Advanced Research Alliance (TARA), University of Tsukuba, Ibaraki, Japan; f International Institute for Integrative Sleep Medicine (WPI-IIIS), University of Tsukuba, Ibaraki, Japan; g Transborder Medical Research Center, Faculty of Medicine, University of Tsukuba, Ibaraki, Japan

**Keywords:** pancreatic *β*-cell, dedifferentiation, MafB

## Abstract

The transcription factor MafB plays an essential role in *β*-cell differentiation during the embryonic stage in rodents. Although MafB disappears from *β*-cells after birth, it has been reported that MafB can be evoked in *β*-cells and is involved in insulin^+^ *β*-cell number and islet architecture maintenance in adult mice under diabetic conditions. However, the underlying mechanism by which MafB protects *β*-cells remains unknown. To elucidate this, we performed RNA sequencing using an inducible diabetes model (*A0B^Δpanc^* mice) that we previously generated. We found that the deletion of *Mafb* can induce *β*-cell dedifferentiation, characterized by the upregulation of dedifferentiation markers, *Slc5a10* and *Cck,* as well as several *β*-cell-disallowed genes, and by the downregulation of mature *β*-cell markers, *Slc2a2* and *Ucn3*. However, there is no re-expression of well-known progenitor cell markers, *Foxo1* and *Neurog3*. Further, the appearance of ALDH1A3^+^ cells and the disappearance of UCN3^+^ cells also verify the *β*-cell dedifferentiation state. Collectively, our results suggest that MafB can maintain *β*-cell identity under certain pathological conditions in adult mice, providing novel insight into the role of MafB in *β*-cell identity maintenance.

## INTRODUCTION

The insulin secretion ability of pancreatic *β*-cells is essential for blood glucose homeostasis maintenance, and the dysfunction of the insulin response is the cause of both forms of diabetes mellitus (type 1 diabetes [T1D] and type 2 diabetes [T2D]). They are characterized by a nearly complete loss of secretory ability that may be due to an autoimmune response or peripheral insulin resistance due to a deficit of serviceable *β*-cells. In addition to *β*-cell death, the dedifferentiation of insulin-secreting *β*-cells has been proposed to be the dominant mechanism underlying functional *β*-cell failure (1). It is characterized by the absence of key maturation marker genes (such as *Slc2a2* and *Ucn3*) and is accompanied by the disruption of key components for insulin secretion (2, 3). In recent years, *β*-cell dedifferentiation has attracted greater attention in both human patients (4, 5) and murine models of T1D and T2D (1, 2). Interestingly, the loss of *β*-cell identity is not unidirectional. Through targeted pharmacological therapy, *β*-cell stress can be alleviated, potentially restoring *β*-cell function and glucose homeostasis (6, 7). However, the mechanisms underlying *β*-cell dedifferentiation are still under investigation.

The term “*β*-cell dedifferentiation” was originally proposed to interpret the loss of the mature *β*-cell phenotype, which is characterized by a regression to a less differentiated or a progenitor-like state (8, 9). However, increasing evidence suggests that the adequacy of dedifferentiation remains debated (10). In contrast, some researchers hold the view that *β*-cell dedifferentiation is the consequence of the disruption of specific genes related to glucose metabolism, the secretory pathway, and protein processing and is accompanied by the upregulation of *β*-cell forbidden genes (11–13) without a regression to a *β*-cell precursor-like stage. Collectively, these alterations result in *β*-cell structural and metabolic reconfiguration as well as disruptive insulin secretion.

MafA and MafB, members of the large Maf subfamily of basic leucine zipper transcription factors, are famous for their exclusive roles in pancreatic *β*-cell development, maturation, and insulin secretion (14). In rodents, due to the unique expression patterns of MafA and MafB, the dominant regulation of *β*-cells in the adult state is mainly affected by MafA (14, 15), indicating the limited function of MafB in *β*-cells after birth. Interestingly, our previous study found that MafB was re-expressed in insulin-secreting cells under MafA-deficient conditions in adult mice (16) and demonstrated that MafB can participate in the functional maintenance of *β*-cells in adult mice under pathological conditions (17). However, the manner in which MafB affects the function of adult *β*-cells is still under investigation.

Following previous results, the present study uses a previously-generated diabetes mellitus murine model of *Pdx1*-dependent, *Mafb*-deletion mice under *Mafa* knockout conditions (*A0B^Δpanc^*) to detect the possible mechanisms of MafB function under pathological conditions in adult mice. By performing a bulk RNA sequencing (RNA-seq) analysis and a comparison with previous reports, such as those of Sachs et al. (7) and Nimkulrat et al. (18), we were able to elucidate the role of MafB in pancreatic *β*-cell function, especially in the maintenance of *β*-cell identity in adult mice.

## RESULTS

### *Mafb* deletion under *MafA*-deficient conditions, impaired glucose tolerance, and abolished insulin secretion ability.

**FIG 1:**
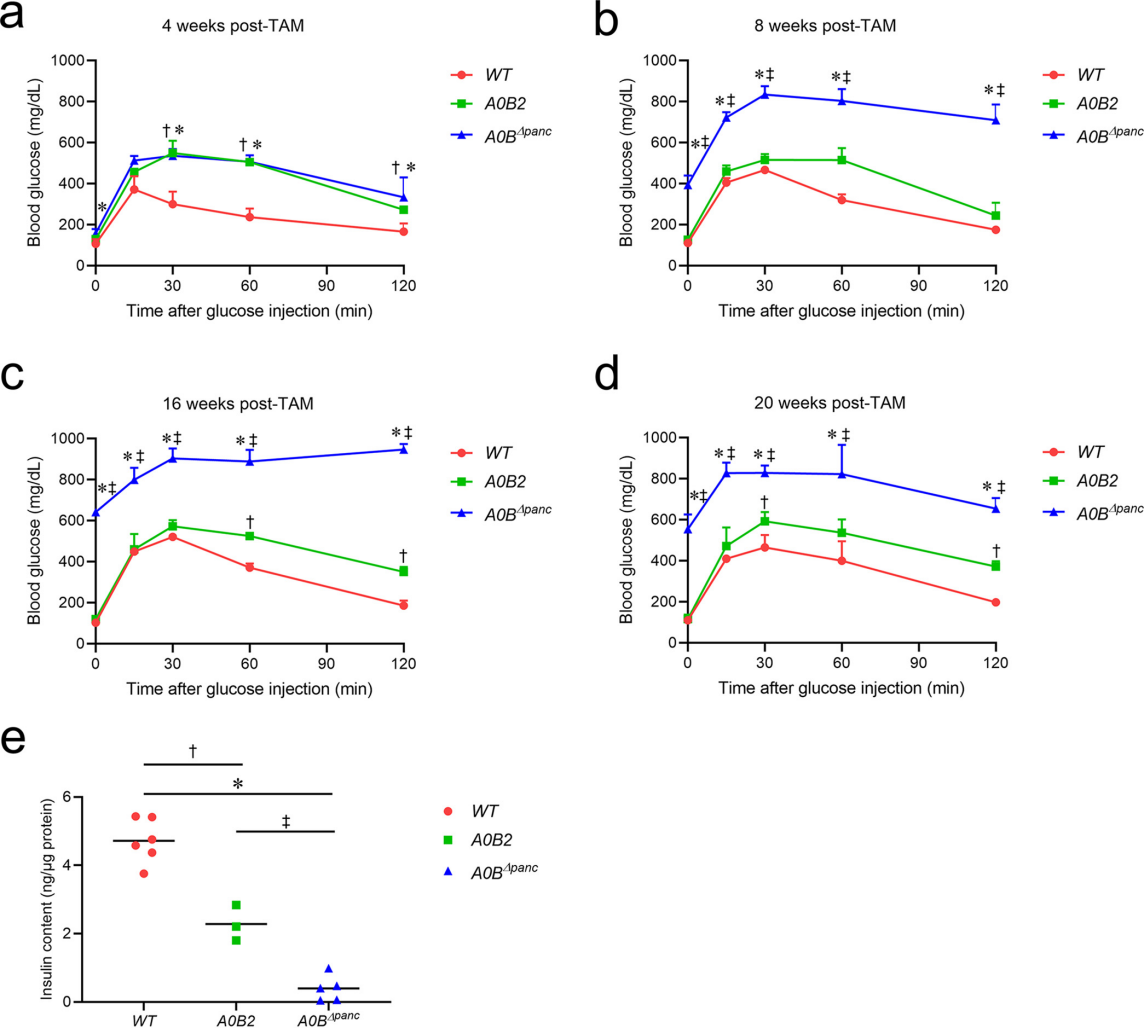
Glucose clearance ability in *A0B^Δpanc^*, *A0B2*, and *WT* mice, measured by an intraperitoneal glucose tolerance test (IPGTT) at (a) 4, (b) 8, (c) 16, and (d) 20 weeks post-TAM (*n* = 3 for each group). (e) Pancreatic insulin content in *A0B^Δpanc^*, *A0B2*, and *WT* mice at 20 weeks post-TAM. Insulin content was normalized to the total protein concentration (*WT*: *n* = 6; *A0B2*: *n* = 3; *A0B^Δpanc^*: *n* = 5). Data are expressed as the mean or the mean ± standard deviation. (*WT versus *A0B^Δpanc^*; *P* < 0.05; ^†^*WT* versus *A0B2*, *P* < 0.05; ^‡^*A0B2* versus *A0B^Δpanc^*, *P* < 0.05).

To determine the optimal time for islet collection and to assess the ability of glucose clearance, the intraperitoneal glucose tolerance test (IPGTT) was implemented to monitor the dynamic changes in blood glucose from 4 to 20 weeks post-tamoxifen (TAM) in *A0B^Δpanc^*. The glucose clearance ability was mildly affected at 4 weeks post-TAM (Fig. 1a). Subsequently, starting from 8 weeks post-TAM, the blood glucose levels in *A0B^Δpanc^* mice were markedly higher than those in *A0B2* and *WT* mice at all of the time points (Fig. 1b to d). The fasting blood glucose levels reached approximately 600 mg/dL from 400 mg/dL after 16 weeks post-TAM, and glucose level peaked at approximately 1,000 mg/dL after 2 h of observation. These data suggest that the deletion of *Mafb* might influence pancreatic *β*-cell function from 4 to 8 weeks post-TAM, and the irreversible repercussion can last at least 20 weeks. Thus, the time for pancreatic islet collection for the following experiments was set at 8 weeks post-TAM.

To check the influence of *Mafb* deletion upon insulin metabolism, the insulin content was measured. The results illustrated that the normalized insulin content was markedly lower in *A0B^Δpanc^* mice compared to those of *WT* and *A0B2* mice. (Fig. 1e, Fig. S1a and S1b). Furthermore, in female mice, the blood insulin levels of each group showed no significant difference before glucose administration. Although both female *A0B2* and *A0B^Δpanc^* mice failed to respond to an elevated glucose level, the circulating levels of insulin were comparable between them (Fig. S1c). On the other hand, after 24 weeks of observation, the body masses of the male *A0B^Δpanc^* mice were significantly lower than those measured in the *WT* and *A0B2* groups from 12 weeks (Fig. S1d). These collective results indicate that the glucose clearance ability was severely diminished, causing hyperglycemia in *A0B^Δpanc^* mice.

### Differentially expressed genes in *A0B^Δpanc^* mice.

**FIG 2:**
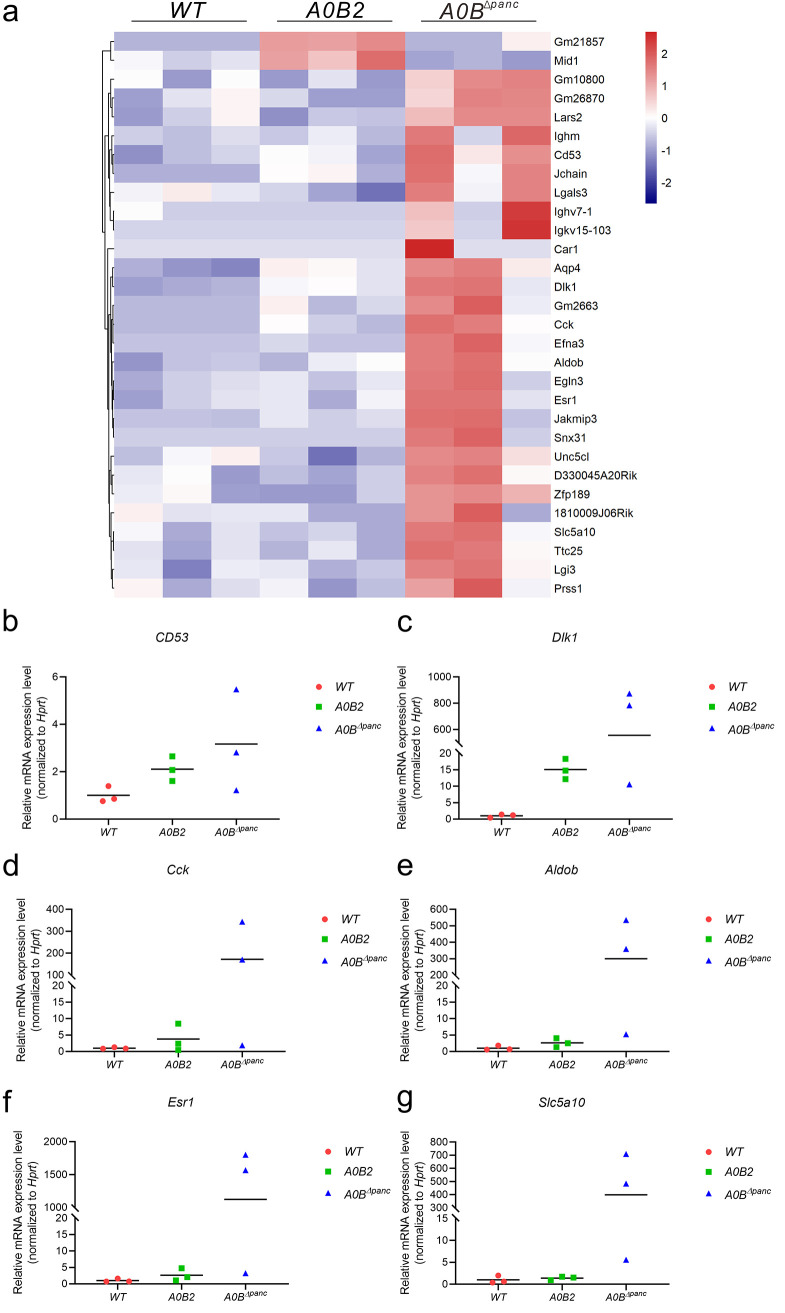
(a) Heat map of differentially expressed genes (DEGs) between *A0B2* and *A0B^Δpanc^* groups (*n* = 3 for *WT*, *A0B2*, and *A0B^Δpanc^*). Color scales depict the normalized expression levels for different genes. Red and blue represent higher and lower values, respectively. (b–g) Relative mRNA expression levels of pro-inflammatory marker *CD53* (Panel b), neonatal *β*-cell marker *Dlk1* (Panel c), and disallowed genes *Cck*, *Aldob*, *Esr1*, and *Slc5a10* (Panel d–g).

To detect the specific functions of MafB under MafA-deficient conditions, we performed RNA-seq using isolated pancreatic islets from *WT*, *A0B2*, and *A0B^Δpanc^* mice and found 30 differentially expressed genes (DEGs) between the *A0B^Δpanc^* and *A0B2* groups (Fig. 2a). Of these, 28 genes were upregulated in the *A0B^Δpanc^* group. Intriguingly, the pro-inflammatory marker *CD53* showed an ascendant trend. Meanwhile, several *β*-cell-disallowed genes (*Dlk1*, *Cck*, *Aldob*, *Esr1*, *and Slc5a10*) were upregulated after *Mafb* conditional knockout. Among them, *Dlk1* was a neonatal *β*-cell marker. Subsequently, the mRNA levels of the target genes were validated by quantitative PCR (qPCR) and found to be consistent with the RNA-seq results (Fig. 2b–g). These results suggest that the functional changes induced by the deletion of *Mafb* may be limited but are specific.

### Pancreatic *β*-cell dedifferentiation induced in *A0B^Δpanc^* mice.

**FIG 3:**
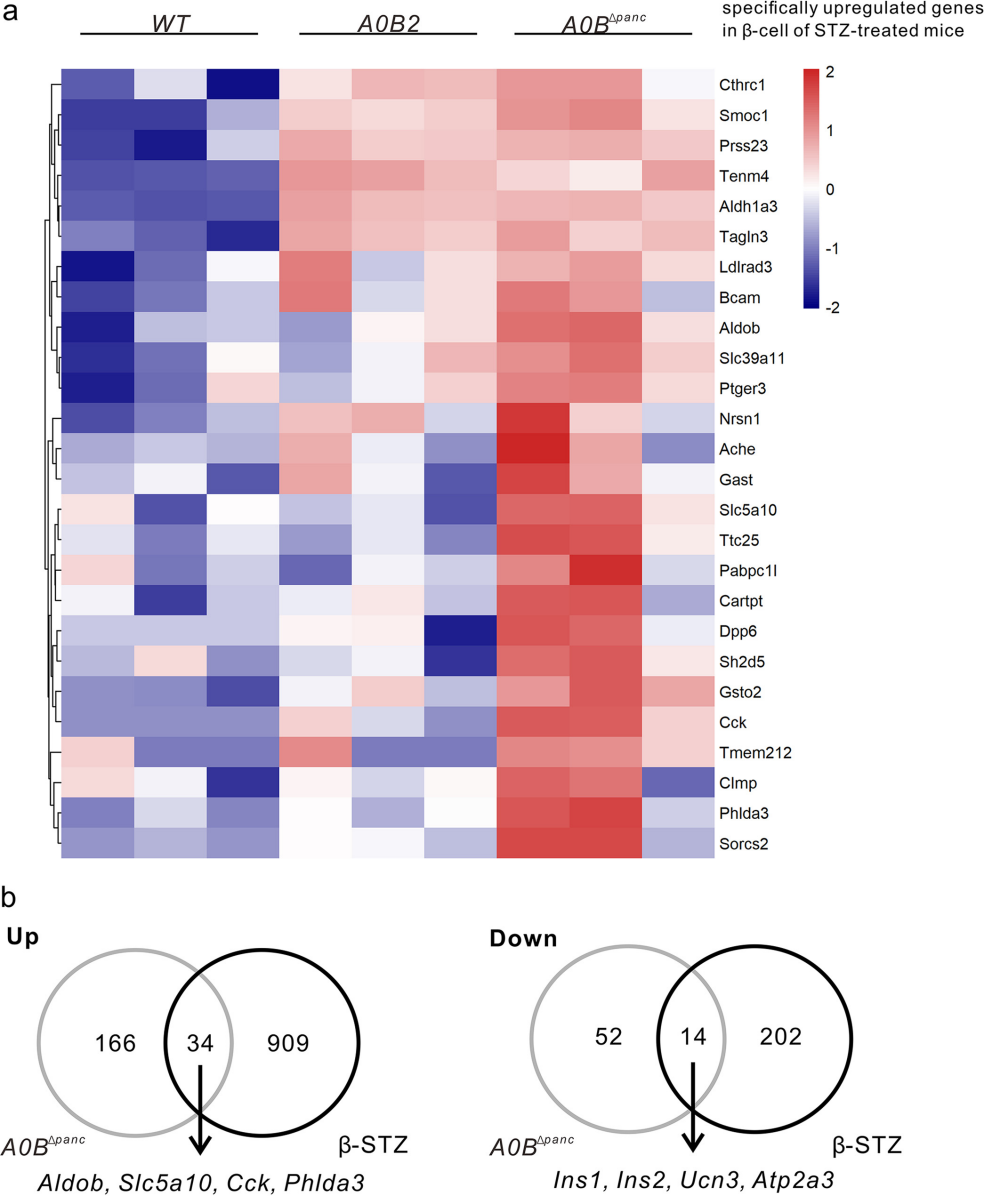
*β*-cell dedifferentiation in *A0B^Δpanc^* mice. (a) Comparison of specifically upregulated genes in *β*-cells of STZ-treated mice. Color scales depict the normalized expression levels for different genes. Red and blue represent higher and lower values, respectively. (b) Comparison between dysregulated genes in *β*-cells in *A0B^Δpanc^* mice and data from scRNA-seq of *β*-cells in STZ-treated mice. Gene names of overlapping markers are listed.

Previously, Sachs et al. (7) generated a mouse model of *β*-cell dedifferentiation by applying multiple low doses of streptozotocin (STZ) (*β*-STZ model). Meanwhile, they proposed two novel markers of *β*-cell dedifferentiation (*Cck* and *Slc5a10*). Both markers showed higher expression levels in *A0B^Δpanc^* mice (Fig. 2). In light of their results, we compared the gene expression profiles between the *β*-STZ model and the *A0B^Δpanc^* mice (Fig. 3). Strikingly, the expression patterns of the *β*-STZ model specifically upregulated genes exhibited similarities to those of the *A0B^Δpanc^* group. Compared to the *WT* group, almost all of the specific genes showed obvious upregulation. Further, the expression values of half of the specific genes were evoked compared to the *A0B2* group, including similarities in *Aldob*, *Slc5a10*, *Cck*, and *Phlda3* (Fig. 3a). To expand the candidate genes that have unique expression patterns in the *A0B^Δpanc^* group, we compared the DEGs from *WT* versus *A0B2* and *WT* versus *A0B^Δpanc^*. Interestingly, the DEGs from *WT* versus *A0B2* were entirely contained in the set of DEGs from *WT* versus *A0B^Δpanc^*, and 266 DEGs induced by *Mafb* deletion were identified (Fig. S2a). Of these, the numbers of upregulated and downregulated genes were 200 and 66. The corresponding enrichment analysis is listed in Fig. S2. After comparison with the gene expression profiles of the *β*-STZ model, an increased expression of genes related to *β*-cell dedifferentiation and insulin secretion (*Aldob*, *Slc5a10*, *Cck*, and *Phlda3*) was common to both the *A0B^Δpanc^* group and the *β*-STZ model. Conversely, a decreased expression of genes involved in *β*-cell maturation and function (*Ins1*, *Ins2*, *Ucn3*, and *Atp2a3*) was common to these two models (Fig. 3b). These results indicate that the deletion of *Mafb* can induce *β*-cell dedifferentiation in *A0B^Δpanc^* mice, which was similar to the results found by the *β*-STZ model.

**FIG 4:**
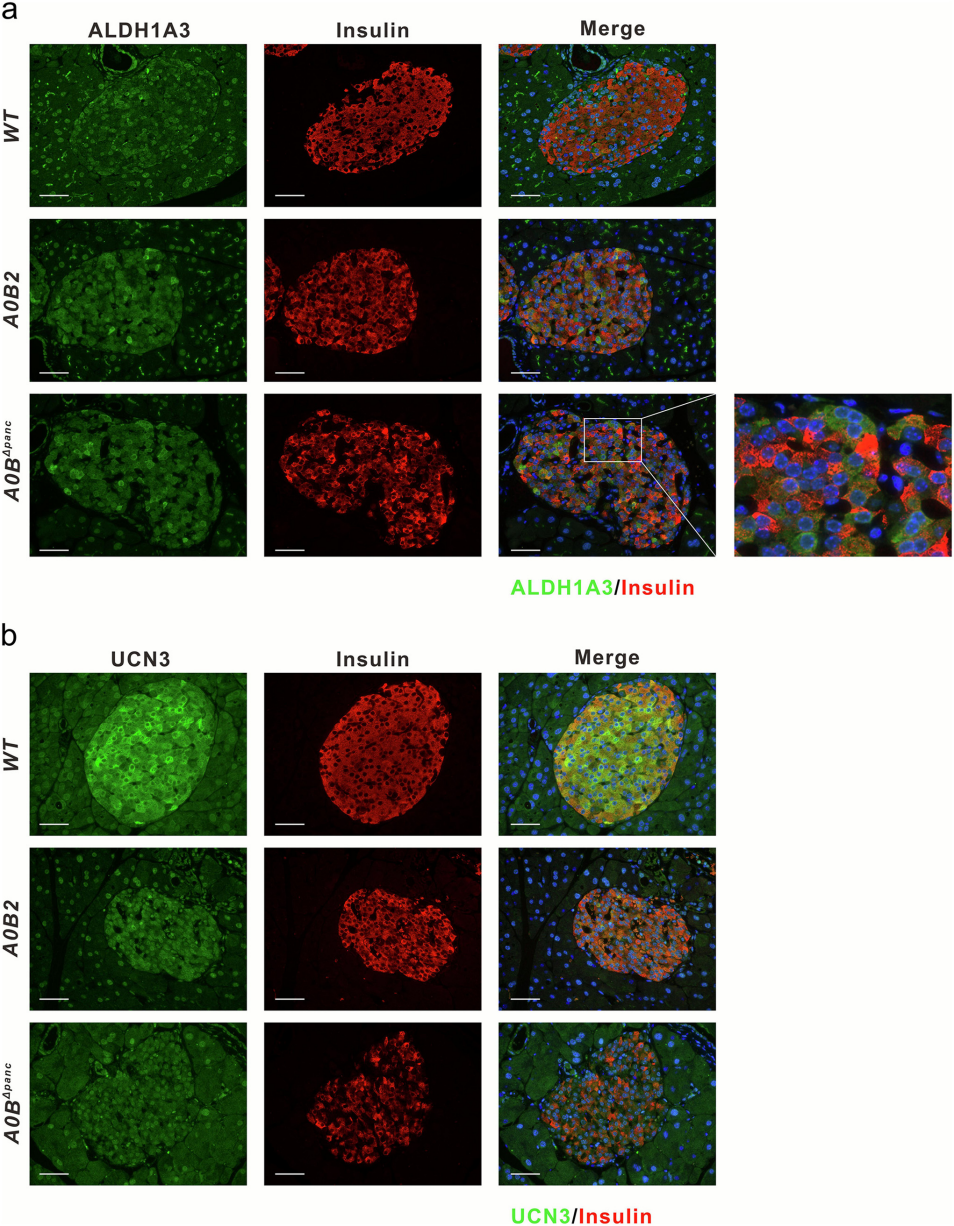
Immunohistochemical validation of *β*-cell dedifferentiation marker ALDH1A3 (a) and maturation marker UCN3 (b). Scale bar = 50 μm.

To validate the *β*-cell state after *Mafb* deletion, two classic markers (ALDH1A3 and UCN3) that indicate the identity of *β*-cells were verified using immunohistochemistry analysis (Fig. 4). As expected, ALDH1A3^+^ cells were rare in the *WT* group. Conversely, the number of ALDH1A3-expressing cells exhibited a considerable and comparable rise between the *A0B2* and *A0B^Δpanc^* groups. Consequently, in the latter group, there were obvious, heterogeneous, positive signals among the ALDH1A3^+^ cells, which showed high levels of ALDH1A3 and low levels of insulin (Fig. 4a). For UCN3, the number was comparable with that of insulin-expressing cells in *WT* group; however, there were few positive cells in the other two groups (Fig. 4b). Collectively, the identities of *β*-cells were strongly affected in both the *A0B2* and *A0B^Δpanc^* groups, and those ALDH1A3-positive cells were comprised of insulin-producing cells.

### The dedifferentiation induced by MafB is not a reversal of the developmental ontogeny.

**FIG 5:**
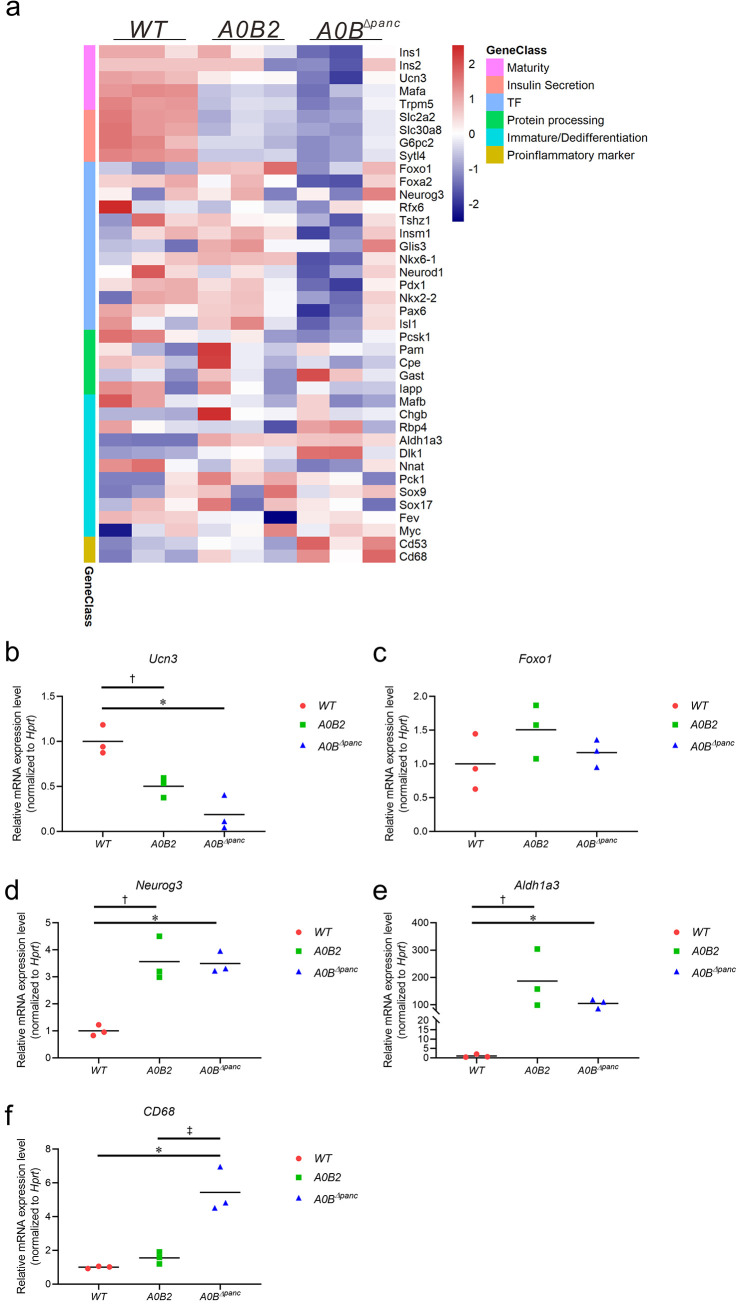
(a) Expression pattern changes of well-known markers of *β*-cell maturity, immaturity, functional maintenance, and crucial transcription factors. Color scales depict the normalized expression levels for different genes. Red and blue represent higher and lower values, respectively (b–f). The relative expression levels of *β*-cell maturation marker *Ucn3* (b), progenitor cell markers *Foxo1* (c) and *Neurog3* (d), dedifferentiation marker *Aldh1a3* (e), and macrophage marker *CD68* (f) are shown. Data are expressed as means (*n* = 3 for *WT*, *A0B2*, and *A0B^Δpanc^*; *WT versus *A0B^Δpanc^*, *P* < 0.05; ^†^*WT* versus *A0B2*, *P* < 0.05; ^‡^*A0B2* versus *A0B^Δpanc^*, *P* < 0.05).

To dissect the possible reasons underlying *β*-cell dedifferentiation, we compared the RNA-seq results with the markers of *β*-cell maturity, insulin secretion, crucial transcription factors (TFs), protein processing, *β*-cell immaturity/dedifferentiation, and pro-inflammatory markers (Fig. 5a). The *β*-cell maturation related genes *Ins1*, *Ins2*, and *Ucn3* were clearly downregulated by *Mafb* deletion (Fig. 5a). Unexpectedly, the well-known *β*-cell progenitor marker genes, such as *Foxo1*, *Rfx6*, *Nkx6-1*, *Neurod1*, and *Pdx1*, did not increase but were slightly downregulated in the *A0B^Δpanc^* group. Similarly, the deletion of *Mafb* did not stimulate the expression of endocrine progenitor cell marker *Neurog3* compared with that of the *A0B2* group. In contrast, the disallowed gene, *Gast*, was highly expressed in *A0B^Δpanc^* mice and was accompanied by *Rbp4* and *Aldh1a3*, two *β*-cell dedifferentiation markers (Fig. 5a). The expression levels of other markers of immature *β*-cells (*Pck1*, *Sox9*, *Sox17*, *Fev*, and *Myc*) were comparable among the *WT*, *A0B2*, and *A0B^Δpanc^* groups. Furthermore, the expression levels of pro-inflammatory markers *CD53* and *CD68* also exhibited obvious upregulation. The relative mRNA expression levels of selected genes were validated by qPCR (Fig. 5b–f). Interestingly, the expression profiles of these marker genes were highly similar to those patterns in the “Anna Karenina model” reported by Nimkulrat et al. (18), possessing no upregulation of *β*-cell progenitor marker genes, thereby indicating that *β*-cell dedifferentiation in *A0B^Δpanc^* mice is not a reversal of the developmental ontogeny.

## DISCUSSION

Talchai et al. found that *β*-cell dedifferentiation is the main cause of diabetic *β*-cell failure (1) and that the prevention or restoration of *β*-cell dedifferentiation is proposed to be a potential method to restore glycemic homeostasis in diabetic patients. Thus, it is necessary to understand the mechanisms that lead to *β*-cell dedifferentiation. The concept of *β*-cell dedifferentiation is used to describe the process of *β*-cell loss, resulting in the disruption of optimal insulin secretion. However, along with the development of diabetic research, the definition of *β*-cell dedifferentiation remains debated (10). Previously, *β*-cell dedifferentiation was mainly described by the loss of *β*-cell maturation markers concomitant with the re-expression of *β*-cell progenitor markers, such as *Neurog3*, *Sox9*, and *Pdx1* (1, 19, 20). However, an increasing number of studies have reported that *β*-cell dedifferentiation involves the upregulation of *β*-cell-disallowed genes as well as the downregulation of *β*-cell-enriched genes (11, 12) without the re-expression of progenitor transcription factors (21–23). In the present study, we conditionally deleted *Mafb* under MafA-deficient conditions. After bulk RNA-seq and comparisons with results from Sachs et al. and Nimkulrat et al. (7, 18), we found that the deletion of *Mafb* can induce *β*-cell dedifferentiation in adult mice. Meanwhile, the appearance of ALDH1A3 and the disappearance of UCN3 stated the *β*-cell identity in the *A0B^Δpanc^* group. However, the dedifferentiation was different from that of previous cases, which reverted to a progenitor-like state by upregulating *β*-cell progenitor genes (such as *Sox9*, *Neurog3*, and *Myc*) (1, 19, 24). Interestingly, mature *β*-cell markers (*Ucn3* and *Slc2a2*) were markedly repressed in *A0B^Δpanc^* mice compared with those in the *A0B2* group, which was concomitant with the upregulation of several *β*-cell-disallowed genes (such as *Dlk1*, *Aldob*, *Slc5a10*, *Rbp4*, and *Aldh1a3*). Strikingly, the “Anna Karenina” model reported by Nimkulrat et al. (18) demonstrates that *β*-cell dedifferentiation is dependent on the degree of the mature identity disruption, resulting in their own, but not reverse-ordered, developmental ontogeny. The similar expression patterns of crucial marker genes and transcription factors between the *A0B^Δpanc^* mice and the “Anna Karenina” model strongly indicates that the *β*-cell dedifferentiation induced by *Mafb* deletion was not a reversal of the developmental ontogeny and occurred through a different process. To the best of our knowledge, this is the first report related to MafB function in *β*-cell identity maintenance in adult mice.

The transcription factor MafB plays an indispensable role in murine *β*-cell development before birth, and its expression is gradually decreased in *β*-cells in adult rodents, whereas MafB is expressed in *β*-cells, even in adult humans. However, our previous study suggested that MafB is involved in *β*-cell maintenance under pathological conditions (16, 17). Here, we demonstrated that dedifferentiation may be one of the reasons for *β*-cell failure under MafA-deficient conditions. Although there was no re-expression of known markers of *β*-cell precursors, the appearance of the disallowed genes may also provide insights into *β*-cell dedifferentiation. For example, *Aldob* is not only negatively associated with insulin secretion in human patients (25) but also involved in mitochondrial dysfunction caused by overproduction of methylglyoxal (26). Intriguingly, both *Slc5a10* and *Aldob* are involved in fructose metabolism and are upregulated in diabetes (27). Furthermore, the upregulation of *Aldh1a3* may also indicate the dysfunction of oxidative phosphorylation and mitochondrial function (28). Thus, it may be reasonable to infer that dedifferentiation is related to mitochondrial metabolism dysfunction.

One of our major drawbacks is that it is still unclear whether the changed expression profiles of the DEGs can be entirely explained by *Mafb* deletion. It is unlikely that the changes induced by glucotoxicity would be similar to those caused by specific gene manipulation, and the interpretation could be complicated. Thus, it is difficult to determine how much of the difference is accounted for by glucotoxicity versus genetic intervention. Interestingly, from the report of Ebrahimi et al., an 11 mg/dL increase of glucose level still could lead to changes in gene expression (29). Thus, it will be intriguing to detect the dynamic changes of blood glucose and gene expression patterns between 4 to 8 weeks post-TAM in a future study. Furthermore, although the normalized count values of *Slc5a10* and *Cck* in the *A0B^Δpanc^* group were significantly higher than those in the *A0B2* group, we could not demonstrate consistent results on mRNA expression levels. This might be explained by the limited number of sequencing samples and inconsistent disease process caused by individual differences, although we verified the genotype and phenotype prior to islet isolation.

Apart from the dedifferentiation marker genes between *A0B^Δpanc^* and *A0B2*, the changes of *CD53*, *CD68*, and *Esr1* were striking enough to draw attention. *CD53*, a member of the tetraspanin superfamily, is highly expressed within different immune cells, indicating there may be inflammation or immune cells infiltration, which may be consistent with the report of Singh et al. (30). Similarly, the increased expression level of macrophage marker *CD68* indicated possible inflammation, as well. As for *Esr1*, it plays a vital role on *β*-cell function maintenance by enhancing glucose-stimulated insulin biosynthesis and protecting *β*-cells from apoptosis (31). Interestingly, a recent study found that estrogen signaling is still required for male mice in the regulation of glucose homeostasis (32). Thus, the mechanisms underlying the induction of *Esr1* mediated by *Mafb* deletion may provide insights on diabetes therapy. Furthermore, as reported, both MafA and MafB are compromised in human T2D *β*-cells (21), which show similar genetic changes compared to those of our model. It will be interesting to determine whether *Mafb* silencing can contribute to *β*-cell identity loss under pathological conditions. In conclusion, MafB can maintain *β*-cell identity under certain pathological conditions, and our results may provide some clues for future research in human diabetics.

## MATERIALS AND METHODS

### Mice.

All the mice used in this study were from the C57BL/6J genetic background and bred under specific-pathogen-free conditions in the Laboratory Animal Resource Center at the University of Tsukuba. All experiments with animals were approved by and conducted in accordance with the guidelines of the University of Tsukuba Animal Ethics Committee (Authorization No. 19-131). In this study, *Mafa*^−/−^;*Mafb*^flox/flox^;*Pdx1*-*CreER*^TM^ (*A0B^Δpanc^*) and *Mafa*^−/−^;*Mafb*^flox/flox^ (*A0B2*) mice were used, and these were generated in a previous study. Briefly, they were generated by crossing *Mafa*^−/−^ with either *Mafb*^flox/flox^;*Pdx1-CreER*^TM^ or *Mafb*^flox/flox^, respectively (33). TAM was administered at 2 weeks of age at a concentration of 75 mg/kg for 5 consecutive days, before intraperitoneal (IP) injection TAM was dissolved in ethanol and then subjected to corn oil (34). The control group included *WT* mice, which were obtained from Japan SLC (Shizuoka, Japan). The body masses of the mice in all three groups were measured and compared at 4, 8, 12, 16, 20, and 24 weeks post-TAM.

### Intraperitoneal glucose tolerance test and insulin content measurement.

The intraperitoneal glucose tolerance test (IPGTT) was performed as previously described (33) at 4, 8, 16, and 20 weeks post-TAM. Briefly, mice were fasted for 16 h, with free access to water. Then, IP injection was administered by using a 20% solution of glucose (2 g/kg body weight). The glucose levels were measured at the designed time points (0, 15, 30, 60, and 120 min after injection) by using a FreeStyle glucometer (Terumo, Tokyo, Japan) from tail vein blood samples.

As for insulin content measurement, whole pancreases were obtained from the *WT*, *A0B2*, and *A0B^Δpanc^* groups at 20 weeks post-TAM. After weighting, they were chopped into small pieces in ice-cold acid-ethanol (1.5% HCl in 75% ethanol). Then, before overnight incubation at 4°C, a 30 s sonication was administered (settings: duty cycle, 20%; output control, 20). On the next day, after a second round of sonication, the supernatants were collected after centrifuging (2400 rpm/min, 30 min, 4°C). Insulin contents were measured by using an enzyme-linked immunosorbent assay (ELISA) (Morinaga mouse insulin ELISA kit; M1102). The obtained results were normalized to the total protein concentration from the collected pancreases, measured by a Coomassie protein assay reagent (Thermo Scientific).

### Pancreatic islet isolation and RNA sequencing.

Pancreatic islets from *WT*, *A0B2*, and *A0B^Δpanc^* mice were isolated as previously described (35). Briefly, prior to inflating the pancreas (digestion by 1 mg/mL collagenase type V, diluted in Krebs-Ringer bicarbonate [KRBH] buffer [129.4 mM NaCl, 5.2 mM KCl, 1.3 mM KH_2_PO_4_, 1.3 mM MgSO_4_, 2.7 mM CaCl_2_, 24.8 mM NaHCO_3_, 10 mM HEPES, pH 7.4]), the common bile duct was clamped. Subsequently, the bloated tissues were subjected to a 20 min incubation at 37°C in a collecting tube with collagenase buffer. Then, the samples were washed with ice-cold KRBH buffer, which contained 0.5% bovine serum albumin (BSA). Finally, the isolated islets were manually picked under a stereomicroscope.

Total RNA was extracted from the collected pancreatic islets from each of the three groups (3 mice from each group) using phenol chloroform extraction (TRIzol, Thermo Fisher Scientific, Waltham, MA, USA). Prior to the construction of the RNA-seq library, rRNA was depleted using the NEBNext rRNA Depletion Kit (New England Biolabs, Ipswich, MA, USA) and subsequently subjected to the NEBNext Ultra Directional RNA Library Prep Kit (New England Biolabs). Libraries were sequenced by NextSeq 500 (Illumina, San Diego, CA, USA) on paired-end mode (2 × 36 bases). The raw sequencing data that passed the quality control were imported into the CLC Genomics Workbench (Version 10.1.1; Qiagen, Hilden, Germany) and mapped to the mouse reference genome (mm10). Gene expression levels were calculated as the values of the total counts and normalized using the quantile method. All sequencing data are available in the Gene Expression Omnibus (GEO) repository under accession number GSE200355.

### Screening for differentially expressed genes and validation.

Differentially expressed genes were screened with total counts greater than 0 by using the Empirical Analysis of DEG tool (edgeR) of the CLC Main Workbench (Version 7.7.3; Qiagen). The DEGs between two groups were selected with FDR-corrected *P* < 0.05. The mRNA expression levels of the target genes were validated by quantitative real-time PCR and relied on Thermal Cycler Dice real-time system (TakaRa) with the SYBR green PCR master mix (TakaRa). The relative expression values were normalized to *Hprt*. All validations were performed in duplicate. The primers used are listed in Table 1.

**TABLE 1:** Primer sequences for real-time quantitative PCR

Gene	Sequences (5′–3′)
*Slc5a10*	F: ATTCAGAAGCGCTTTGGTGG
	R: AATGGTGTACAGGGCCGTAA
*Cck*	F: ACTGCTAGCGCGATACATCC
	R: AAATCCATCCAGCCCATGTA
*Ucn3*	F: CAGAGGACCTGCAGGGAGTA
	R: GAACTTGTGGGAGAGGCTTG
*Aldob*	F: CACACAGCTTCTGATACCTTGG
	R: TGAGCCATGATGACAGGTACA
*Esr1*	F: TCTCCATGATCAGGTCCACC
	R: AGATCTCCACCATGCCTTCC
*Dlk1*	F: GCAGTGCATCTGCAAGGAT
	R: GTCCACGCAAGTTCCATTGT
*Aldh1a3*	F: CCCACGGTCTTCTCAGATGT
	R: TCACCTCCTCCAGGTTTTTG
*CD53*	F: GCAGTGTTGTGGTGTAAATGG
	R: GAAATTGGAGTGAAACCACGA
*CD68*	F: TCTCTAAGGCTACAGGCTGCT
	R: CAATGATGAGAGGCAGCAAG
*Neurog3*	F: ACTGACCTGCTGCTCTCTATTCTTT
	R: GGCGCCATCCTAGTTCTCC
*Foxo1*	F: TCACCCTGTCGCAGATCTAC
	R: GTTGTTGTCCATGGACGCAG

### Pancreatic immunohistochemistry analysis.

Owing to the limited number of samples, the 21-week-old pancreata from the 5 weeks TAM injection group (33) were used for pancreatic immunohistochemistry analysis. Briefly, the collected tissues were fixed in 4% (wt/vol) paraformaldehyde (PFA) overnight. After embedding in paraffin, tissues sliced with 2-μm-thickness were subjected to blocking in donkey serum for 1 h at 37°C, followed by heat-induced epitope retrieval by retrieval solution (Dako, pH = 6). Rabbit anti-urocortin III (UCN3) (1:200, Phoenix Pharmaceutical), rabbit anti-ALDH1A3 (1:200, Novus) and guinea pig anti-insulin (1:100, Abcam) were used as primary antibodies. After 16 h of incubation at 4°C, antigens were visualized after incubation with species-specific secondary antibodies conjugated with Alexa Fluor 488 or 594 (1:800, Life Technologies). Nuclei were marked by Hoechst 33342 (Molecular Probes). After mounting, the signal visualization was achieved under a fluorescence microscope (BiorevoX-800, Keyence).

### Gene functional analysis.

To detect the possible functional changes involved in the DEGs caused by *Mafb* deletion, Metascape (36) (https://metascape.org/gp/index.html#/main/step1) was used for genetic enrichment analysis.

### Statistical analyses.

All data are presented as means or means and standard deviations (SD). The comparisons between two groups and among three groups were performed using Student's *t* tests, and one-way analyses of variance (ANOVA) with *post hoc* Tukey’s honestly significant difference (HSD) tests, respectively, with multiple biological replicates. Statistical significance was set at *P* < 0.05.

### Data and resource availability.

All data supporting the conclusions are presented in the main text and supplemental material. The next-generation sequencing data generated in this study have been submitted to the NCBI Gene Expression Omnibus (GEO: http://www.ncbi.nlm.nih.gov/geo/) under accession number GSE200355.
