# Supplementary material for: MafB Maintains β-Cell Identity under MafA-Deficient Conditions
Source: Mol Cell Biol. 2022 Jul 11;42(8):e00541-21. doi: 10.1128/mcb.00541-21 (PMC9387222; doi:10.1128/mcb.00541-21)
Supplement: Supplemental file 1 — Fig. S1 and S2. Download mcb.00541-21-s0001.pdf, PDF file, 0.3 MB [file mcb.00541-21-s0001.pdf]

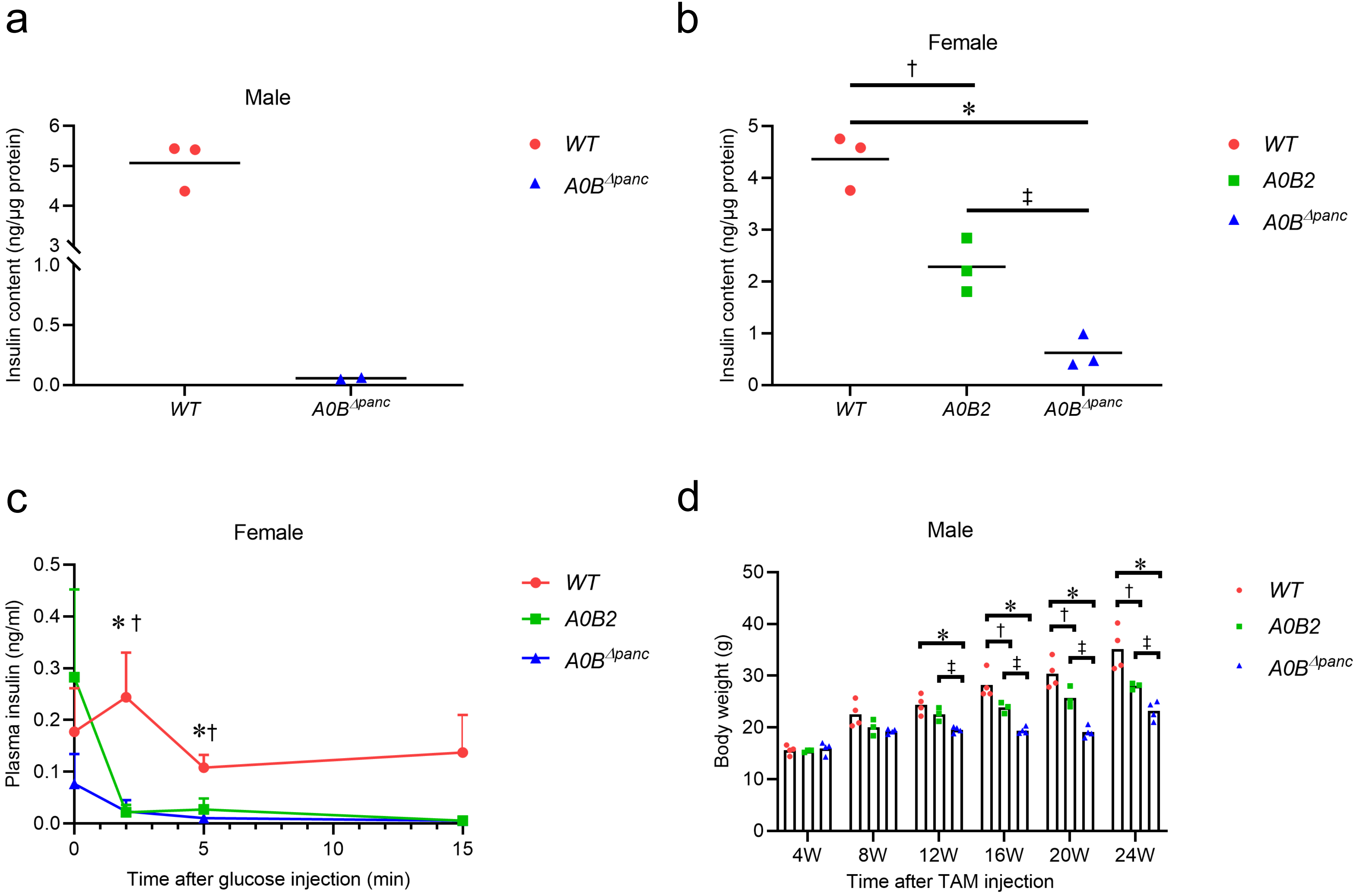

Fig S1 (a and b) Pancreatic insulin content in both male (a; *WT*, n = 3; *A0B*$^{\Delta panc}$, n = 2) and female (b; *WT*, n = 3; *A0B2*, n = 3; *A0B*$^{\Delta panc}$, n = 3) mice at 20 weeks post-TAM. Insulin content was normalized to the total protein concentration. (c) In vivo GSIS test after intraperitoneal loading with glucose (3 g/kg) was performed on 24 weeks post-TAM female mice from *WT* (n = 3), *A0B2* (n = 3), and *A0B*$^{\Delta panc}$ (n = 3) groups. (d) Changes in the body weight of *WT* (n = 4), *A0B2* (n = 3) and *A0B*$^{\Delta panc}$ (n = 4) mice from 4-24 weeks post-TAM. Data are expressed as mean ± SD or mean. (*, *WT* vs *A0B2*, $P < 0.05$; †, *WT* vs *A0B*$^{\Delta panc}$, $P < 0.05$; ‡, *A0B*$^{\Delta panc}$ vs *A0B2*, $P < 0.05$.)

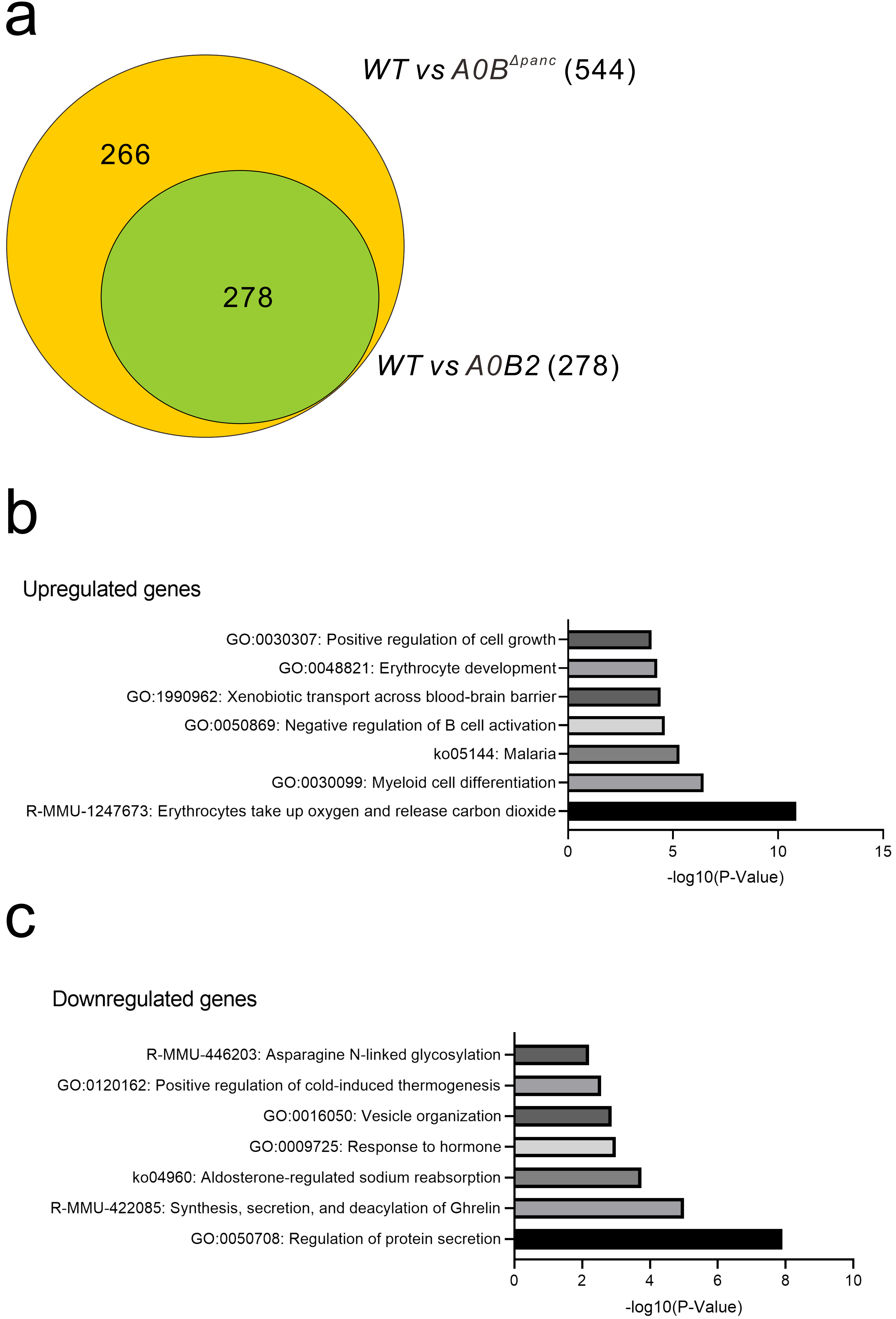

Fig S2 (a) The scheme for screening DEGs induced by *Mafb* deletion. (b and c) Enrichment analysis of upregulated and downregulated genes from *Mafb* specific DEGs.
